# Catheter ablation of atrial fibrillation using 2nd-generation cryoballoon in congenital heart disease patients — significance of RF ablation of additional atrial macro-reentrant tachycardia

**DOI:** 10.1007/s10840-022-01213-0

**Published:** 2022-04-13

**Authors:** Ulrich Krause, Matthias J. Müller, Heike E. Schneider, Thomas Paul

**Affiliations:** Department of Pediatric Cardiology and Intensive Care Medicine, University Medical Center, Georg-August-University Göttingen, Robert-Koch-Str. 40, 37099 Göttingen, Germany

**Keywords:** Atrial fibrillation, Congenital heart disease, Cryoballoon, Pulmonary vein isolation, Atrial tachycardia

## Abstract

**Background:**

Prevalence of atrial fibrillation (AF) is increasing in adult patients with congenital heart disease (CHD). Experience using the cryoballoon to achieve pulmonary vein isolation (PVI) in adult CHD patients is limited. The aim of the present study was to assess the value of PVI by cryoballoon in adult CHD patients and to evaluate the significance of additional radiofrequency (RF) ablation of atrial tachycardia (AT).

**Patients and methods:**

Prospective data analysis; all patients with CHD and AF and PVI using the cryoballoon from January 2017 through November 2021 were included.

**Results:**

Nineteen patients with various types of CHD were included. Median age was 58 (IQR 47–63) years. A total of 12/19 (63%) patients had had RF ablation of right atrial AT before. Median procedure duration was 225 (IQR 196–261) min. Median fluoroscopy time was 12.3 (IQR 5.2–19.5) min and median freeze time was 32 (IQR 28–36.3) min. Procedural success was achieved in all patients. Additional RF catheter ablation of intraatrial reentrant tachycardia within the left atrium was performed in 3/19 (16%) subjects and within the right atrium in 6/19 (32%) patients. Median follow-up was 26 (IQR 9–49) months. Excluding a 90-day blanking period, recurrence of AF was observed in 6/19 subjects (32%). After one redo procedure deploying RF energy only, 84% of all patients remained free from recurrence. Phrenic nerve palsy was observed in 1 subject.

**Conclusion:**

Results after PVI using the cryoballoon plus additional RF ablation of AT were promising (84% success including one redo procedure). Success of AF ablation was unsatisfactory in all patients who had no additional AT ablation. Ablation of any AT in these patients should therefore be considered in addition to PVI.

## Introduction

Arrhythmias are a significant issue in the aging adult congenital heart disease population (ACHD) [1]. Atrial fibrillation (AF) is diagnosed with increasing prevalence in adults with congenital heart disease (CHD), most often decades after corrective surgery. AF is the second most common atrial tachyarrhythmia following intraatrial reentrant tachycardia (IART) observed in this unique population. Prevalence of AF in ACHD patients increases with age and in those aged > 50 years, AF is the most common atrial tachyarrhythmia [2]. In contrast to IART, AF is not associated with complexity of CHD but with patient’s age and blood pressure [2]. As factors such as sinus node dysfunction or ventricular dysfunction limit the use of antiarrhythmic medications and results of antiarrhythmic drug therapy are often discouraging in ACHD patients, catheter ablation of AF has been suggested as treatment option for ACHD patients by a recent consensus statement [1]. The results of radiofrequency (RF) catheter ablation of AF in ACHD patients were promising with midterm success rates comparable to subjects without CHD [3–5]. Despite growing evidence on the value of AF ablation in the ACHD population, data is still sparse and most studies report experience on RF catheter ablation of AF in those patients. Even less evidence is available on the significance of AF ablation in ACHD patients using the recently introduced cryoballoon [6–8]. Ablation of AF using the cryoballoon has been demonstrated non-inferior to RF ablation in a recent randomized trial on individuals without CHD [9]. In order to increase the body of knowledge on the significance of cryoballoon ablation of AF in adult patients with CHD, the aim of the present study was to assess the value of pulmonary vein isolation (PVI) by cryoballoon in ACHD patients with AF and to evaluate the significance of additional RF ablation of IART.

## Patients and methods

All subjects with CHD who had catheter ablation of AF deploying the 2nd-generation cryoballoon (Arctic Front Advance™, Medtronic, Minneapolis, USA) between January 2017 and November 2021 were prospectively enrolled at the time of EPS and ablation. The study was approved by the institutional review board and fully complies with declaration of Helsinki. Prior to EPS and ablation, written informed consent was obtained from all patients.

All patients had either paroxysmal AF or persistent AF without evidence for primary macro-reentrant atrial tachycardia degenerating into AF. In subjects with primary macro-reentrant atrial tachycardia degenerating into AF, EPS and ablation of atrial tachycardia were performed as previously described [10]. Individuals with permanent AF [11] were excluded from the study.

### EPS and ablation

EPS and ablation were performed at a tertiary ACHD center highly experienced in mapping and ablation of atrial tachycardia (AT) and AF in patients with CHD. All procedures were performed by a single operator (UK) and under general anesthesia. Antiarrhythmic medication was discontinued at least 5 half-lives prior to EPS. If patients were in AF at the beginning of the procedure, electrical cardioversion into sinus/atrial rhythm or atrial paced rhythm was performed. Complete hemodynamic assessment and detailed angiographies including selective coronary angiography were performed during the same procedure if data had not been obtained within the last 6 months prior to the procedure. In order to rule out intraatrial thrombi, transesophageal echocardiography was performed prior to EPS and ablation.

Venous vascular access was obtained via the femoral vessels in all patients. An arterial sheath (5F) was introduced into either the right or left femoral artery or the right radial artery (at discretion of the operator) for retrograde left heart access in order to perform systemic ventricular angiography, for coronary angiographies and for continuous blood pressure monitoring. Transseptal access to the left atrium was gained using either a Fast Cath™ SL1 sheath (8 F, Abbott, Abbott Park, USA) or a steerable long sheath (Agilis™ NxT, 8.5 F, Abbott, Abbott Park, USA) and a standard Brockenbrough needle (either 89 or 98 cm, Brockenbrough BRK™, Abbott, Abbott Park, USA). After obtaining transseptal access, heparin was administered as a bolus infusion of 100 IU/kg body weight (maximum 5000 IU heparin) and then as continuous infusion of initially 10.000 IU/h in order to maintain the activated clotting time (ACT) at 300–350 s. ACT was regularly checked every 20 min and heparin infusion was adjusted accordingly. For pacing maneuvers and as reference for activation mapping, a decapolar electrode catheter was placed within the coronary sinus (5F, Livewire™, Abbott, Abbott Park, USA).

Pulmonary vein isolation was achieved using the second-generation cryoballoon (Arctic Front Advance™, 23 and 28 mm, Medtronic). Balloon diameter was chosen according to the diameter of the pulmonary vein ostia (as measured by angiography or computed tomography). The cryoballoon catheter was advanced to the pulmonary vein ostia using a deflectable Flex Cath™ Advance sheath (15 F outer diameter, Medtronic, Minneapolis, USA), which was (in exchange for the sheath used for transseptal puncture) advanced to the left atrium. Signal recording from and stimulation within the pulmonary vein ostia was performed using the Achieve Advance™ mapping catheter (15 mm, 20 mm, Medtronic, Minneapolis, USA). Cryoablation was performed for the duration of 240 s/PV ostium.

During cryoablation of the right pulmonary vein ostia, phrenic nerve function was monitored continuously by stimulation of the phrenic nerve via a quadripolar electrode catheter (6 F, Supreme™, Abbott, Abbott Park, USA) placed within the superior vena cava. In case of loss of phrenic nerve capture during ablation, freezing was immediately stopped.

Programmed atrial stimulation and atrial burst stimulation were performed in all patients after completion of PVI. Additional RF catheter ablation was performed in all patients with documented atrial tachycardia in addition to AF (either on Holter ECG or on pacemaker memory) or in those subjects with inducible sustained atrial macro-reentrant tachycardia on programmed atrial stimulation or atrial burst stimulation respectively. In cases with additional RF catheter ablation, a force sensing TactiCath SE™ ablation catheter (8F, Abbott, Abbott Park, USA) was used. RF ablation was performed in a temperature-controlled mode with a target temperature of 45 °C and the generator energy output set at 30 W. Target catheter contact force was 10–20 g and target lesion size index (LSI) was > 5. Mapping of atrial macro-reentrant tachycardia was performed using the EnSite Precision™ system (Abbott, Abbott Park, USA) using a high-density multipolar mapping catheter (Advisor™ HD Grid, Abbott, Abbott Park, USA). If the EnSite Precision™ system was applied, it was used to guide the Achieve Advance™ mapping catheter into the pulmonary veins in order to reduce the use of fluoroscopy and to precisely obtain a voltage map prior and after PVI. After AF ablation, all patients were put on oral anticoagulation with either vitamin K antagonists or direct oral anticoagulants. Depending on CHA_2_DS_2_-VASc-Score, oral anticoagulation was continued for at least 3 months after ablation or indefinite.

Recurrence of AF was any documented episode of paroxysmal or persistent AF (either symptomatic or asymptomatic) after catheter ablation.

All patients were followed regularly at our adult congenital heart disease outpatient clinic at intervals of 1 and 6 months after ablation. Further follow-up was every 12 months, or every 6 months in patients with cardiac implantable electronic devices (CIED; pacemaker or implantable cardioverter defibrillator).

### Statistics

Statistical analysis was performed using SPSS® 27.0 software (IBM, Armonk, USA). Numerical data are presented as median and interquartile range (IQR) unless otherwise indicated. Differences in categorical variables were calculated by the chi-square or Fisher’s exact test where appropriate. Differences in parametric data were calculated by the Kruskal-Wallis test. Statistical significance was set at *p*<0.05.

## Results

### Patients

A total of 19 patients were enrolled into the study between January 2017 and November 2021. Nine out of nineteen (47%) individuals were female. Median age at the time of ablation was 58 (IQR 47–63) years and median body weight was 86.6 (IQR 78–95) kg. Median body mass index was calculated with 28.7 (IQR 25.6–34.3) kg/m^2^.

Patients enrolled into the present study suffered from various types of congenital heart disease. Table 1 gives detailed information on the congenital cardiac conditions. Fifteen out of nineteen (79%) subjects had undergone surgery for their congenital cardiac condition during childhood. A total of 3/19 patients presented with native CHD without surgical or catheter intervention so far (secundum type atrial septal defect *n*=1, partial anomalous venous drainage *n*=1, Ebstein’s anomaly *n*=1), and one patient had had catheter-based stent implantation for coarctation of the aorta at the age of 55 years. A total of 12/19 (63%) patients had had RF ablation of right atrial AT including induction of bidirectional conduction block within the cavotricuspid isthmus before. Five out of nineteen (26%) patients had a dual-chamber pacemaker implanted. Indication for pacemaker implantation was sinus node dysfunction (*n* = 4) and postoperative complete AV block (*n* = 1). A total of 4/19 (21%) subjects had a dual-chamber cardioverter-defibrillator (ICD) implanted for primary prevention of sudden cardiac death. Paroxysmal AF was diagnosed in 13/19 (68%) individuals and persistent AF was present in the remaining 6/19 (32%) patients.

**Table 1:** Types of the congenital heart defect of the patients enrolled; 16/19 subjects had had prior repair of their cardiac condition (right column).

CHD type	*n* (total)	*n* (s/p repair)
AS, MR	1	1
ASD II	2	1
AVSD	3	3
CoA	2	2
CoA, VSD	1	1
DCRV	2	2
DORV, ccTGA, situs inversus, VSD	1	1
M. Ebstein	1	0
MS	1	1
PAPVD	2	1
SVAS, WBS	1	1
TOF	2	2
Total	19	16

(*ASD II*, secundum type atrial septal defect; *AS/MR*, valvular aortic stenosis/mitral regurgitation; *AVSD*, atrioventricular septal defect; *CoA*, coarctation of the aorta; *DCRV*, double chambered right ventricle; *DORV/ccTGA*, double outlet right ventricle/congenitally corrected transposition of the great arteries; *M. Ebstein*, Ebstein’s anomaly; *MS*, congenital mitral stenosis; *PAPVD*, partial anomalous pulmonary venous drainage; *SVAS*, supravalvular aortic stenosis; *TOF*, tetralogy of Fallot; *VSD*, ventricular septal defect; *WBS*, Williams-Beuren syndrome)

### Procedural characteristics

Median procedure duration was 225 (IQR 196–261) min and median fluoroscopy time during EPS and PVI was 12.3 (IQR 5.2–19.5) min. A three-dimensional electroanatomical mapping system (3D-EAM) was used for mapping and catheter navigation in 13/19 (68%) subjects. Using 3D-EAM, fluoroscopy time could be significantly reduced. Median fluoroscopy time was 20.8 (IQR 12.1–32.5, *n*=6) min in cases performed without the application of 3D-EAM and 5.5 (IQR 4.1–13.7, *n*=13) min in cases performed with 3D-EAM (*p*=0.03). Procedure duration was not different with the use of 3D-EAM (207 min, IQR 166–240, *n*=6 without 3D-EAM vs. 232 min, IQR 208–272 min with 3D-EAM, *p*=0.17). At the beginning of the procedure, 12/19 (63%) patients were either in normal sinus rhythm or spontaneous atrial rhythm (*n*=10) or in atrially paced rhythm (*n*=2). The remaining 7/19 (37%) subjects were in AF. Electrical cardioversion was performed after induction of anesthesia and after intraatrial thrombus formation had been ruled out by transesophageal echocardiography. Median overall freeze time was 32 (IQR 28–36.3) min.

### Procedural success

Procedural success (defined as electrical isolation of all pulmonary vein ostia and ablation of all inducible macro-reentrant tachycardias) was achieved in all patients. In 2/19 (10%) subjects, additional RF ablation of the lower right pulmonary vein was required as it was impossible to achieve adequate occlusion of the lower right pulmonary vein ostium with the cryoballoon. Additional RF ablation of intraatrial reentrant tachycardia (IART) within the left atrium was performed in 3/19 (16%) subjects and within the right atrium in 6/19 (32%) patients, respectively. In total, 9/19 (47%) patients required additional RF ablation of intraatrial (macro-) reentrant tachycardia; one subject had right atrial as well as left atrial RF ablation within a single procedure. Intraatrial macroreentrant circuits were the only atrial tachycardias targeted in the present study. The likelihood of the need for additional RF ablation was neither associated with the type of AF (paroxysmal vs. persistent, *p*=0.14) nor with age (*p*=0.54). However, considering the type of congenital heart disease, subjects with left obstructive lesions (like coarctation of the aorta) and those with repaired left-to-right shunt lesions were significantly more prone to require additional RF ablation (*p*=0.009).

### Recurrence and follow-up

All patients were followed at the ACHD outpatient unit of our institution. Median follow-up time was 26 (IQR 9–49) months. One subject suffered from sudden cardiac death during follow-up. This particular patient with double outlet right ventricle and congenitally corrected transposition of the great arteries had repeatedly refused ICD implantation for primary prevention of sudden cardiac death. This particular patient had recurrence of AF 8 months after the primary ablation procedure and deceased before redo procedure could be performed.

**Fig. 1:**
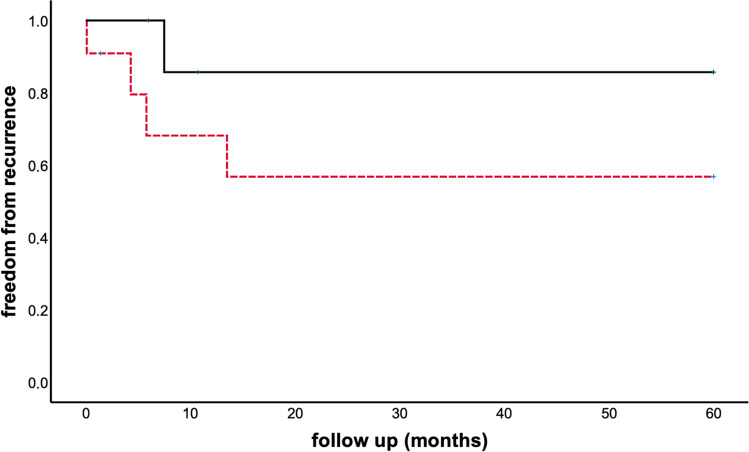
Kaplan-Meier curve of AF recurrence after pulmonary vein isolation (PVI) only using the cryoballoon only (*dashed red line*) and after PVI with cryoballoon and additional radiofrequency ablation of atrial tachycardias (*solid black line*)

**Fig. 2:**
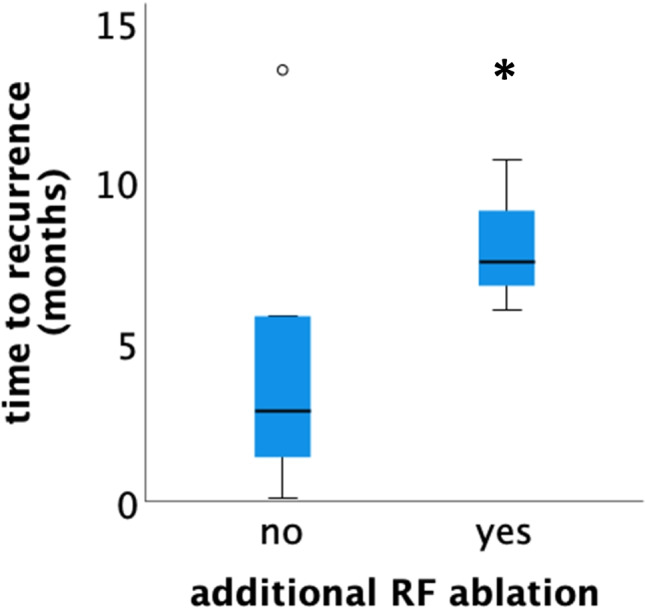
Time to recurrence was significantly longer after pulmonary vein isolation with cryoballoon and additional radiofrequency ablation of atrial tachycardia (*n* = 3, *asterisk*, *p*=0.048) compared to cryoablation of pulmonary vein ostia alone (*n* = 6)

Recurrence of AF was observed in a total of 9/19 subjects (47%) after the initial procedure. Excluding a 90-day blanking period after the initial procedure, 6/19 patients (32%) had recurrence of AF with a median time to recurrence of 203 (IQR 163–342) days after cryoenergy PVI +/− additional RF ablation. Considering all 9 subjects with recurrence of AF, there was a trend towards fewer recurrences after additional RF ablation (either RA or LA) as recurrence of AF occurred in 3/8 (37%) subjects after additional RF ablation compared to 6/11 (55%) individuals with cryoballoon PVI only (Fig. 1; *p* = 0.46). Time to recurrence was significantly longer in patients with cryoballoon ablation and additional RF ablation of AT (Fig. 2; median time to recurrence 7 months, *n* = 3 vs. 3 months, *n* = 6; *p* = 0.048). There was no association of type of AF (paroxysmal vs. persistent) and incidence of AF recurrence (*p* = 0.87). Prior repair of the underlying CHD was not associated with freedom from recurrence of AF (*p*=0.9). Interestingly, the use of 3D-EAM was significantly associated with efficacy (*p*=0.009).

In 7/9 (78%) subjects with initial PVI +/− additional RF ablation of AT and recurrence of AF, redo procedures aiming for identification and ablation of conduction gaps within the pulmonary vein ostia and/or atrial substrates were performed. Median time to first redo procedure was 383 (IQR 178–455) days. In 2/19 (11%) individuals, reconnection of PV was documented during a redo procedure. One subject with Ebstein’s anomaly had reconnection of the right inferior as well as the left inferior PV; in this subject, cryoballoon PVI without additional RF ablation of IART was performed during the initial procedure. Another patient with aortic stenosis and mitral regurgitation had reconnection of the right inferior PV as well as reconnection in the area of left superior PV/left atrial appendage ridge. This particular patient had had additional RF ablation of IART within the LA during the initial procedure. In the remaining 5 redo procedures, recurrent IART were targeted in 2 subjects (right atrial and left atrial substrates in one individual each) and in the remaining 3 patients, new IART were targeted within the left atrium. In all redo procedures, RF ablation exclusively was performed. Mapping and focal RF ablation of conduction gaps were performed in all individuals with reconnected pulmonary veins. After one redo procedure, recurrence of AF was observed in 3/19 (16%) of all patients. In summary, after initial cryoballoon PVI +/− additional RF ablation and one redo procedure using RF energy to either close conduction gaps within the pulmonary vein ostia or for ablation of atrial macro-reentrant tachycardia, 84% of all patients remained in SR/spontaneous atrial rhythm/atrial paced rhythm during follow-up.

Electrical cardioversion of AF during follow-up was significantly less required after the first catheter ablation compared to prior to ablation (*n*=6/19, 32% post ablation versus 18/19, 95% prior ablation, *p*=0.028).

### Complications

In 1 patient (5%), right-sided phrenic nerve palsy was observed during follow-up. In this particular subject (63 years old female after interventional treatment of coarctation of the aorta), phrenic nerve stimulation had been performed during cryo-ablation of the right-sided pulmonary vein ostia as in all other patients. This particular patient presented with two separate right pulmonary vein ostia and a 23-mm Arctic Front Advance™ cryoballoon (Medtronic) had been used. Cryoablation had immediately been stopped after loss of phrenic nerve capture. On follow-up visit 4 weeks later, the patient still presented with phrenic nerve palsy. However, she did not complain about shortness of breath or other symptoms attributable to phrenic nerve palsy. No case of atrioesophageal fistula was observed.

## Discussion

AF represents a constantly growing issue in the aging ACHD population and treatment concepts for this unique population of patients have to be developed. For the adult population without congenital heart disease, catheter ablation of AF by electrical isolation of the pulmonary vein ostia using either RF energy or cryoenergy has been recommend by a recent guideline document [12]. PVI using the cryoballoon has been shown to be non-inferior to RF ablation of the pulmonary vein ostia and superior to drug therapy alone in patients with paroxysmal AF [9, 13]. Also in patients with persistent AF, PVI applying the cryoballoon showed favorable results in subjects with structurally normal hearts [14]. However, despite robust evidence for PVI using the cryoballoon in the general population, there is less evidence for its role in the treatment of AF in the aged ACHD population, though recent reports demonstrated feasibility and usefulness of the cryoballoon in ACHD patients [6, 8].

The present study addresses the role of PVI using the cryoballoon and the significance of additional AT ablation in the treatment of paroxysmal and persistent AF in a cohort of aged ACHD patients. The main results were [1] despite good initial procedural results (primary endpoint reached in all patients), recurrence rate of AF was considerable after the primary procedure deploying the cryoballoon +/− RF ablation of AT and [2] after a second procedure using RF to close conduction gaps within the pulmonary vein ostia and aiming for ablation of atrial macroreentrant tachycardia, freedom from AF recurrence was achieved in 84% of all patients. Though statistically not significant, there was a trend towards a better outcome after additional RF ablation of AT during the initial procedure.

Comparing the present data with results from a recent single-center study on AF ablation using RF only in adult subjects with CHD [5], our results deploying the 2nd-generation cryoballoon +/− additional RF ablation of macroreentrant AT are similar with respect to recurrence rates after a primary ablation procedure as well as with respect to freedom from AF including redo procedures during follow-up. It is of note that PV reconnection occurred in only 10% of patients in the present cohort, which is a lower connection rate compared to previously published data from patients with structurally normal hearts [15]. Our data was not able to confirm recent data, suggesting a higher PV reconnection rate in subjects requiring touch up RF ablation to achieve complete PVI after cryoballoon ablation as none of our patients presenting with PV reconnection initially required touch up RF ablation to achieve PVI [16].

Time to recurrence was significantly longer in those subjects with additional RF ablation of AT, underscoring the need for mapping and ablation of AT in addition to PVI. The fact that recurrence of AF was less likely using 3D-EAM is most likely attributable to the better and more precise mapping of atrial fibrosis and atrial activation during AT with 3D-EAM. Left atrial posterior wall isolation (LAPWI) in addition to PVI was not routinely performed in the present study but seems to be of significance as a recent report showed good results of PVI in combination with LAPWI in ACHD patients [8]. In subjects lacking structural heart disease, LAPWI was shown to be without any additional benefit [17].

Results imply that ablation of atrial substrates and/or atrial macroreentrant tachycardias in addition to PVI is of significance in ACHD patients with AF compared to the general population. What is the most likely explanation for that? Longstanding atrial pressure and/or volume-overload results in fibrosis of atrial myocardium, which ultimately serves as substrate for AT and AF [18]. Therefore, it may be speculated that besides triggers from the pulmonary veins also atrial substrates promote and maintain AF in ACHD patients and therefore need to be targeted during ablation procedures for AF.

Present data nicely advocate the use 3D-EAM not only for mapping of atrial fibrosis and atrial activation during AT but also as a tool to significantly reduce radiation exposure during cryoballoon PVI. One of the major drawbacks of cryoballoon ablation is the longer fluoroscopy time and the use of contrast dye required to reach the procedural endpoint compared to RF ablation using 3D EAM. Previous studies demonstrated the value of 3D-EAM use in combination with cryoballoon PVI [19] which is confirmed by the present study.

Complication rate in the present study was within the expected range. Phrenic nerve palsy is an issue of concern using the cryoballoon for PVI and occurred in one patient while applying cryoenergy to the upper right pulmonary vein ostium. This complication has been reported to occur in up to 5% of all patients after cryoballoon ablation. Phrenic nerve palsy after cryo-ablation is usually transient and resolves spontaneously within 12 months in most of the cases [20].

## Limitations

The present study is mainly limited by its single-center design, the number of patients enrolled, and the heterogeneity of types of underlying CHD. Though non-inferiority of cryoballoon-PVI has been shown in the general AF population [9], the present study was insufficient to analyze the significance of cryoballoon-PVI compared to RF-PVI in the aged ACHD population with AF.

## Conclusion

AF is of growing concern in the aging ACHD population. PVI using the cryoballoon offers an alternative to PVI with RF energy and can effectively be used also in patients with ACHD. However, as ACHD patients commonly present with different/additional substrates for AT and AF compared to subjects with structurally normal hearts, mapping and ablation of additional atrial macroreentrant tachycardias in combination with PVI seem to be of paramount importance in order to achieve best long-term results. As robust statements on the significance of PVI +/− additional ablation of atrial tachycardias can only be made after analyzing larger patient cohorts, prospective multicenter trials on catheter ablation of AF in the ACHD population will be needed.
